# Impact of Left Atrial Hemodynamics on the Development of Pulmonary Vein Stump Thrombus: Results of Early and Late Postoperative Studies

**DOI:** 10.5761/atcs.oa.25-00099

**Published:** 2026-01-09

**Authors:** Tadashi Umehara, Takuya Tokunaga, Koji Takumi, Go Kamimura, Masaya Aoki, Kazuhiro Ueda

**Affiliations:** 1 Department of General Thoracic Surgery, Kagoshima University Graduate School of Medical and Dental Sciences, Kagoshima, Kagoshima, Japan; 2 Department of Radiology, Kagoshima University Graduate School of Medical and Dental Sciences, Kagoshima, Kagoshima, Japan

**Keywords:** pulmonary vein stump thrombus, 4D flow MRI, lung cancer, hemodynamics

## Abstract

**Purpose:**

Pulmonary vein stump thrombus (PVST) is a relatively common complication after left upper lobectomy that can cause vital organ embolism. We previously found that patients with PVST on postoperative day 7 show risky hemodynamic features around the pulmonary vein stump on 4-dimensional (4D) flow magnetic resonance imaging (MRI), which may contribute to thrombus development. However, it remains unclear whether such hemodynamics persist later.

**Methods:**

Eleven patients who underwent left upper lobectomy for lung cancer received 4D flow MRI on postoperative day 7 and again after over 3 months. Hemodynamic parameters were used to classify each case as risky or non-risky for PVST.

**Results:**

According to a total of 24 examinations in 11 patients, 7 were classified as risky and 17 as non-risky. PVST developed in 6 patients during various postoperative phases, and all PVST cases developed under the risky conditions. Furthermore, PVST did not develop under non-risky conditions, suggesting that our risk assessment is valid as a predictive marker for PVST.

**Conclusion:**

Our results suggest that late postoperative hemodynamic assessments, as well as early postoperative assessments, are useful for identifying patients at high risk of PVST. A late postoperative hemodynamic assessment may contribute to determining when to discontinue anticoagulants.

## Introduction

Pulmonary vein stump thrombus (PVST) is a potentially serious postoperative complication that can lead to vital organ embolization.^1)^ Although the overall incidence of PVST is 3.3%–5.0%, most thrombi develop in the left superior pulmonary vein stump after left upper lobectomy, with an incidence of 13.5%–17.9%.^2–7)^ Several risk factors for PVST formation have been reported, such as division of the pulmonary vein with a stapling device, a longer residual pulmonary vein stump, and impaired left atrial function.^2,6,8–11)^ However, the exact physiological mechanisms underlying PVST remain unknown. Furthermore, although PVST develops predominantly early postoperatively, it can also develop late postoperatively.^12)^

Once PVST is identified on enhanced computed tomography (CT), anticoagulant therapy is generally recommended to prevent vital organ embolisms. However, PVST can recur after its disappearance following discontinuation of the anticoagulant. The reason why the nature of PVST is poorly understood may be due to the limited modalities available for accurately evaluating left atrial hemodynamics, except for transesophageal echocardiography. To clarify the hemodynamic features underlying PVST after lung lobectomy, we preliminarily performed 4-dimensional (4D) flow magnetic resonance imaging (MRI) 7 days after lung lobectomy for primary lung cancer, which does not require contrast materials or radiation exposure. As a result, we found a specific hemodynamic environment, with special reference to blood flow variability, around the pulmonary vein stump after left upper lobectomy, while it was rarely found after resection of other lobes, which may explain the high prevalence of PVST after left upper lobectomy.^7)^ We further evaluated the differences in hemodynamic features between patients who developed PVST after left upper lobectomy and those who did not, and clarified that patients with PVST show specific hemodynamic features regarding blood flow variability and blood flow energy change.^13)^ We therefore focused on 2 quantitative parameters obtained from 4D flow MRI. The standard deviation (SD) of flow velocity reflects temporal variability of local blood flow during a single cardiac cycle and thus serves as a marker of flow instability and oscillatory shear stress at the vein–atrium junction.^13)^ The energy loss (EL) represents viscous dissipation caused by turbulence, vortex formation, and flow separation,^13,14)^ which may impair effective washout of the vein stump and promote endothelial injury. In our previous study of early postoperative patients, PVST cases clustered around specific ranges of SD and EL,^13)^ suggesting that these parameters together characterize a hemodynamic milieu that favors thrombus formation. Building on these findings, we incorporated SD and EL into a composite risk score to stratify PVST risk in early postoperative phases.^13)^

In this study, we hypothesized that SD and EL would be useful in identifying patients at a high risk of PVST in the late phase after left upper lobectomy. If our hypothesis is verified, anticoagulants can be reasonably administered to patients for an appropriate period. To verify our hypothesis, we performed late-postoperative 4D flow MRI in patients who underwent a left upper lobectomy.

## Materials and Methods

### Patients

The present study included 11 patients who underwent left upper lobectomy for lung cancer with radical intent at our institution from July 2019 to June 2022. To exclude the effect of variations in the cardiac function on pulmonary venous flow, patients with the following conditions were excluded: significant asynergy in the left ventricle, ejection fraction <50%, atrial fibrillation, and grade ≥III mitral valve regurgitation. The study was approved by our institutional review board (No.190286E), and informed consent was obtained from all patients.

4D flow MRI was performed on postoperative day 7, and further 4D flow MRI was performed when the patient visited our institution during the postoperative follow-up. Patient characteristics are shown in **Table 1**.

**Table 1:** Patient characters according to the presence of pulmonary vein thrombus

Variables	Total	PV thrombus formation		p Value
		Present (N = 6)	Absent (N = 5)	
Demographic factors				
Age (years)	72.7 ± 5.8	73.3 ± 7.1	72.4 ± 5.5	0.85
Gender (M/F)	5/6	3/3	2/3	1
Body mass index (kg/m^2^)	21.6 ± 2.4	22.0 ± 3.2	21.4 ± 2.0	0.74
Underlying conditions				
Diabetes mellitus (yes/no)	2/9	2/4	0/5	0.445
Preop. antithrombotics (yes/no)	3/8	2/4	1/4	1
Hematological parameters				
WBC count (/mm^3^)	5023 ± 1618	5498 ± 1842	4452 ± 1247	0.294
Hemoglobin (g/dL)	11.9 ± 2.7	11.2 ± 2.8	12.7 ± 2.6	0.37
Platelet count (×10^4^/mm^3^)	20.3 ± 6.5	19.3 ± 8.6	21.5 ± 3.3	0.59
PT-INR	0.96 ± 0.07	0.97 ± 0.08	0.96 ± 0.07	0.759
D-dimer (µg/mL)	1.72 ± 2.37	2.67 ± 2.96	0.60 ± 0.39	0.149
Fibrinogen (mg/dL)	335 ± 129	365 ± 146	276 ± 71	0.263
Cardiopulmonary function				
FEV1/FVC (%)	74.8 ± 10.9	75.2 ± 14.9	74.3 ± 4.3	0.892
%DLCO (%)	98.6 ± 24.7	105.1 ± 31.4	90.8 ± 17.5	0.371
LVEF (%)	66.7 ± 6.5	66.7 ± 8.0	66.7 ± 5.9	0.995
MR (grade 0/1/2)	5/6/0	3/3/0	2/3/0	1
CHA_2_DS_2_-VASc score	2.6 ± 1.1	3.00 ± 1.095	3.2 ± 1.924	0.843

CHA_2_DS_2_-VASc stroke score: congestive heart failure, hypertension, age ≥75 years (doubled), diabetes mellitus, prior stroke or transient ischemic attack (doubled), vascular disease, age 65–74 years, female sex; WBC: white blood cell; PT-INR: prothrombin time-international normalized ratio; FEV1/FVC: forced expiratory volume in 1 s to forced vital capacity ratio; DLCO: diffusing capacity of the lung for carbon monoxide; LVEF: left ventricular ejection fraction; MR: mitral valve regurgitation

### Operation

Left upper lobectomy was performed under general anesthesia with endobronchial intubation by open thoracotomy (n = 5), video-assisted thoracoscopic surgery (n = 3), or robot-assisted thoracoscopic surgery (n = 3). An endoscopic stapler (Ethicon, Cincinnati, OH, USA; Covidien, Minneapolis, MN, USA) was used to divide the pulmonary artery, pulmonary vein, bronchus, and fused fissures. Proximal ligation of the left superior pulmonary vein before stapling was performed in 9 patients.

### 4D flow MRI

To calculate the parameters obtained from blood flow velocity and cine MRI, analysis was performed using iT Flow (Cardio Flow Design Inc., Tokyo, Japan). Cross-sectional flow velocities at the junction of the pulmonary vein stump and left atrium were measured, and changes in flow velocity in each section were evaluated from the instantaneous flow velocity at multiple time points during a single heartbeat. The distance to the pulmonary vein stump was measured in the coronal section. The plane velocity at the junction of the pulmonary vein stump and the left atrium was measured, and its SD was extracted to determine the change in velocity within 1 heartbeat. We also measured the EL, which is reported to be an indicator of turbulence,^14)^ in the entire pulmonary vein stump. We have previously developed the following regression equation using SD and EL for assessment of the risk of PVST early postoperatively^13)^:

$$\begin{array}{rl} \text{Regression equation}=\; & 0.753-0.182\times \left|\text{SD}-3.290\right|\\ & -0.123\times \left|\text{EL}-1.147\right| \end{array}$$

The SD and EL were measured in the early and late postoperative phases of the 11 patients in this study, and a risk assessment derived from regression equations was performed at all time points. Based on the cutoff value of 0.227, which was determined using a receiver-operating characteristic (ROC) analysis in our previous study, the patient was considered to be at high or low risk of PVST.

To assess measurement reproducibility, intra-observer variability was evaluated in a subset of 7 randomly selected cases. Each case was reanalyzed by the same operator after an interval of more than 2 weeks, using identical segmentation planes and analysis thresholds. The intraclass correlation coefficients (2-way mixed model, absolute agreement) were 0.833 for SD and 0.879 for EL, indicating good reproducibility of these parameters.

### The diagnosis of PV thrombus

PVST was first detected using cine MRI. It has been reported that cine MRI can detect left atrial/left atrial appendage thrombi with high accuracy.^15,16)^ In addition, all PVSTs were confirmed by enhanced CT when the thrombus was noted on cine MRI.

### Statistical analyses

Values are expressed as the mean ± SD. To compare patients with and without PVST, an unpaired t-test was used for continuous variables of clinical and 4D flow parameters (SD and EL). The area under the ROC curve (AUC) was calculated to assess the risk of PVST using various parameters. Statistical significance was set at p <0.05. Statistical analyses were performed using the SPSS software (SPSS Statistics version 26; IBM, Chicago, IL, USA).

## Results

**Fig. 1:**
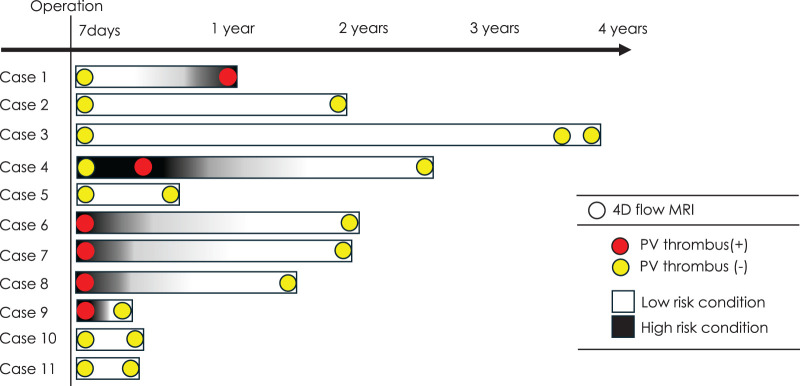
Time course of the hemodynamic condition around the left superior pulmonary vein stump on 4D flow MRI (high-risk condition or low-risk condition) in 11 patients who underwent left upper lobectomy. Pulmonary vein stump thrombus was found only in situations with a high-risk condition on 4D flow MRI and was not found in any situations with a low-risk condition. However, PV thrombus was not found in 1 patient with a high-risk condition (day 7 in Case 4). These findings suggest the validity of late-postoperative 4D flow MRI. 4D: 4-dimensional; MRI: magnetic resonance imaging; PV: pulmonary vein

On postoperative day 7, the hemodynamic condition was diagnosed as risky by 4D flow MRI in 5 of the 11 patients (**Fig. 1**). In these 5 high-risk patients, PVST was identified in 4 patients (Cases 7–10) but not in 1 patient (Case 4). Four patients with PVST received anticoagulant therapy, which resulted in the disappearance of PVST. Subsequent 4D flow MRI revealed that a non-risky hemodynamic condition in all 4 patients. Interestingly, 1 patient without PVST, irrespective of the risky condition, subsequently developed PVST, which was also treated with anticoagulant therapy, resulting in the resolution of PVST and a shift to a non-risky the hemodynamic condition (Case 4). In contrast, the hemodynamic condition on postoperative day 7 was diagnosed as non-risky in the remaining 6 patients. This remained non-risky in these patients, except for 1 patient whose condition later became risky and who developed PVST as late as 14 months after left upper lobectomy (Case 1). Fortunately, vital organ embolisms did not develop in 11 patients throughout the observation period. Eventually, 6 patients experienced PVST at some postoperative time point, while the remaining 5 patients did not. Anticoagulation therapy was administered to the 6 patients with documented PVST (Cases 1, 4, 6, 7, 8, and 9). Five patients (Cases 1, 4, 6, 8, and 9) received direct oral anticoagulants, and 1 received warfarin (Case 7). Treatment was initiated immediately after detection of PVST and continued for 3 months to 2 years, with discontinuation determined by thrombus resolution confirmed on enhanced CT. In all treated cases, normalization of hemodynamic parameters on 4D flow MRI coincided with thrombus resolution. Recurrence of PVST was not detected after discontinuation of anticoagulation therapy. These observations suggest a potential role for serial hemodynamic assessment in tailoring the duration of anticoagulation.

The characteristics of the patients with and without PVST are shown in **Table 1**. There were no significant differences in various preoperative factors, including age, sex, underlying disease, and blood examination results, between the groups. The CHA_2_DS_2_-VASc score (congestive heart failure, hypertension, age ≥75 years [doubled], diabetes mellitus, prior stroke or transient ischemic attack [doubled]; vascular disease, age 65–74 years, female sex), known as a risk parameter of cardiogenic cerebral infarction,^17)^ was also not markedly different between the groups. Regarding intraoperative factors, the surgical approach, surgery time, and amount of bleeding were not significantly different between the 2 groups. In contrast, the pulmonary vein stump length, which is known to be a risk factor for PVST, was longer in patients with any PVST than in those without it (18.8 ± 2.9 vs. 13.8 ± 3.5, p = 0.0356; **Table 2**).

**Table 2:** Surgical factors according to the presence of pulmonary vein thrombus

Variables	Total	PV thrombus formation		p Value
		Present (N = 6)	Absent (N = 5)	
Approach (open/VATS/RATS)	5/3/3	3/2/1	2/1/2	1
Time (min)	261 ± 63.3	261.5 ± 75.3	260.2 ± 54.2	0.974
Blood (mL)	155 ± 128	160 ± 151	149 ± 112	0.897
Ligation (Y/N)	9/2	5/1	4/1	1
PV stump length (mm)	16.51 ± 3.99	18.79 ± 2.88	13.8 ± 3.5	0.0356

VATS: video-assisted thoracic surgery; RATS: robot-assisted thoracic surgery; PV stump length: pulmonary vein stump length

**Fig. 2:**
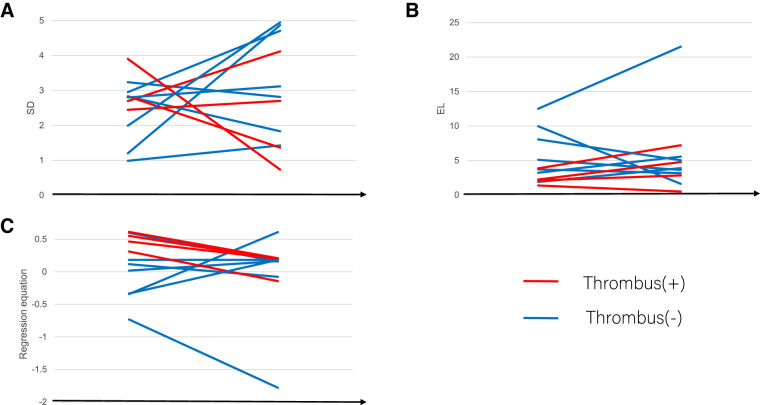
Changes in the SD (**A**), EL (**B**), and risk equation (**C**) between early- and late-postoperative cases. The values for SD, EL, and the risk equation changed from early to late postoperatively in overall patients without a significant trend according to paired t-test findings. Likewise, the values for SD, EL, and the risk equation changed from early- to late-postoperatively when analyses were restricted to patients with PVST, as well when the analysis was restricted to patients without PVST, except that the risk equation decreased from the early postoperative period to the late postoperative period in all patients with PVST (paired t-test, p <0.001). EL: energy loss; PVST: pulmonary vein stump thrombus; SD: standard deviation of flow velocity

**Figure 2** shows the change in the SD, EL, and risk equation between the early and late postoperative periods. The values for the SD, EL, and the risk equation changed from early to late postoperatively in all patients without a significant trend in the paired *t*-test. Likewise, the values for SD, EL, and the risk equation changed variably from early to late postoperatively when the analysis was restricted to patients with PVST, as well as when the analysis was restricted to patients without PVST, except that the risk equation decreased from the early postoperative period to the late postoperative period in all patients with PVST (paired t-test, p <0.001).

**Fig. 3:**
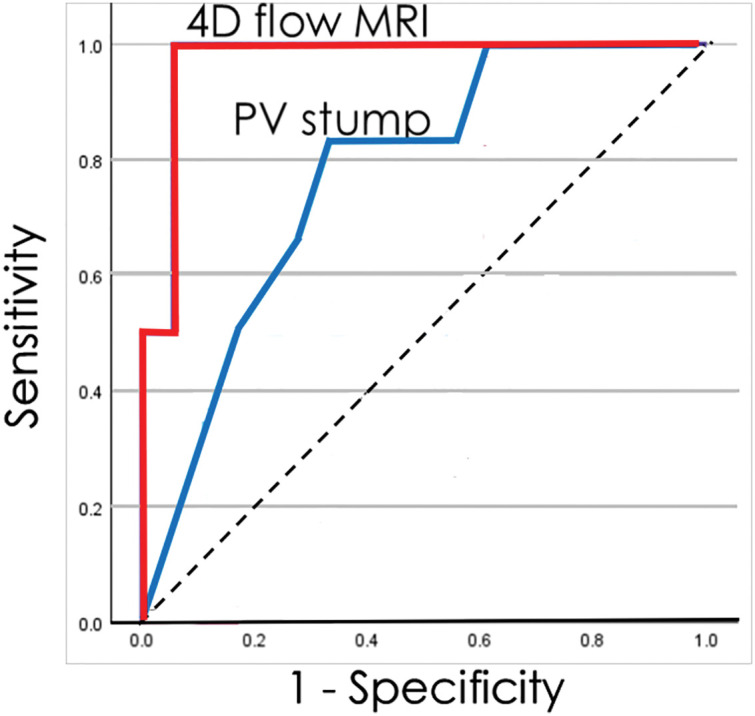
According to the ROC analysis to clarify the potential of diagnosing the risk of PVST, the AUC was 0.773 (95% CI: 0.528–0.956) for the length of pulmonary vein stump and 0.972 (95% CI: 0.899–1.000) for the risk equation. AUC: area under the curve; CI: confidence interval; PVST: pulmonary vein stump thrombus; ROC: receiver-operating characteristic

Eventually, 24 hemodynamic conditions were measured in 11 patients during the observation period. Among the 24 examinations, PVST was detected in 6 and not detected in 18. When 4D flow MRI-derived hemodynamic parameters were compared between the 6 and 18 examinations, EL, |SD-3.290|, |EL-1.147|, and the risk equations were significantly different between the groups (**Table 3**). According to the ROC analysis to clarify the potential of diagnosing the risk of PVST, the AUC was 0.773 (95% confidence interval [CI]: 0.528–0.956) for the length of the pulmonary vein stump and 0.972 (95% CI: 0.899–1.000) for the risk equation (**Fig. 3**). Regarding the risk equation, the best cutoff value in the ROC analysis was 0.311. The sensitivity, specificity, positive predictive value, negative predictive value, positive diagnostic value, and false negative value were 0.311, 100%, 94.4%, 85.7%, 100%, 95.8%, and 0%, respectively.

**Table 3:** MRI-derived parameters according to the presence of pulmonary vein thrombus

Variables	Total	PV thrombus formation		p Value
		Present (N = 6)	Absent (N = 18)	
SD (mL/s)	2.589 ± 1.100	2.824 ± 0.576	2.511 ± 1.230	0.412
EL (10^−3^mW)	4.984 ± 4.493	2.291 ± 0.876	5.882 ± 4.865	0.0073
|SD − 3.290| (mL/s)	1.070 ± 0.725	0.669 ± 0.231	1.204 ± 0.787	0.0177
|EL − 1.147| (10^−3^mW)	3.891 ± 4.444	1.144 ± 0.876	4.807 ± 4.790	0.00576
Regression equation	0.0794 ± 0.5110	0.4902 ± 0.1228	−0.0574 ± 0.5191	0.000456

EL: energy loss; SD: standard deviation for low blood flow rate; Regression equation = 0.753 − 0.182 × |SD − 3.290|−0.123 × |EL − 1.147|

## Discussion

Although the overall incidence of cerebral infarction after lung lobectomy for primary lung cancer is around 1%,^18,19)^ comparable to that after surgery for digestive tract cancer, the incidence becomes higher, reaching up to 4.5% when the analysis is restricted to patients undergoing left upper lobectomy.^6,8)^ This finding is consistent with the high prevalence of PVST after left upper lobectomy. Although surgeons have attempted to reduce the incidence of PVST by ligating the proximal side of the superior pulmonary vein stump followed by dividing the pulmonary vein with a stapler, PVST still develops in a proportion of patients.^9,13,20)^ In addition, the timing of PVST development varies among individuals, and it can develop days^21)^ or years after lobectomy.^12)^ We previously reported that patients with PVST 7 days after left upper lobectomy showed specific hemodynamic features around the pulmonary vein stump on 4D flow MRI.^13)^ However, the validity of 4D flow MRI findings in the later postoperative period (>7 postoperative days) remains unknown because the hemodynamics around the pulmonary vein stump can change with the anatomical rearrangement of the heart and the remaining lung lobe in a time-dependent manner. In the current study, we analyzed data from 4D flow MRI taken on various postoperative days and clarified that 4D flow MRI-based assessment of hemodynamics around the pulmonary vein stump is useful for distinguishing patients with and without PVST, suggesting that 4D flow MRI can help identify patients at high risk of PVST requiring anticoagulant therapy.

The regression equation consisted of 2 parameters: EL and SD. SD represents the degree of change in blood flow velocity around the pulmonary vein stump, and EL represents the loss of blood flow energy. The EL of the flow is caused by the loss of power, such as when passing through complicated units.^14,22)^ The EL arises from turbulence, surface friction, and flow attachment. The lost energy may be transformed into other types of energy, such as heat and energy that wears away at the vascular inner surfaces. The clinical usefulness of EL has been validated for predicting the outcomes of cardiovascular surgery.^23,24)^ In our previous study, patients with PVST were concentrated in a certain area of the scatterplot of SD and EL. Therefore, we considered that the median values of the SD and EL in patients with PVST may be the ideal hemodynamic condition for developing PVST, with a median SD of 3.290 and a median EL of 1.147. In contrast, we considered that an area far from the coordinate (SD = 3.290, EL = 1.147) may be a safe hemodynamic condition that is difficult for PVST to develop. Thus, a regression equation (0.753 – 0.182 × |SD – 3.290| – 0.123 × |EL – 1.147|) was generated from the regression analysis. In a previous study, the AUC for the regression equation diagnosing PVST on postoperative day 7 was 0.918.^13)^

Virchow described 3 critically important factors in the development of venous thrombosis: blood stasis, activation of blood coagulation, and venous intimal damage.^22)^ In the current study, patients with atrial fibrillation, which likely causes left atrial blood stasis, were not included because such patients may have a high risk of PVST as well as a high risk of left atrial appendage thrombus. However, we believe that simple blood stasis cannot explain the high prevalence of PVST after left upper lobectomy but not after other types of lobectomies. As mentioned above, unique hemodynamic conditions, such as blood turbulence, are believed to be associated with the development of PVST. An additional reason for excluding patients with atrial fibrillation is that 4D flow MRI is applicable only to patients with regular sinus rhythm, not patients with atrial fibrillation.

PVST generally develops early postoperatively, from the day of surgery to several months after surgery. Most cases of early postoperative PVST resolve after anticoagulant therapy and rarely recur after discontinuation of the therapy. The findings shown in **Fig. 1** are concordant with this observation. In addition, **Table 3** suggests that the risky condition, which we originally proposed in our former study with early postoperative patients, can also be applied to late postoperative patients. The results of the current study suggest that an assessment of hemodynamic conditions around the pulmonary vein stump is helpful in identifying patients who should receive prophylactic anticoagulants, as well as in identifying patients in whom anticoagulant therapy should be stopped (as was suggested by Case 4). Interestingly, there was an exceptional case in which the hemodynamic condition changed from non-risky to risky, resulting in the development of PVST late postoperatively (Case 1). However, such an event has been reported in clinical practice. These exceptional cases highlight the dynamic nature of hemodynamic risk and emphasize the importance of longitudinal surveillance, as well as the current limitations in predictive sensitivity. Thus, although the classification of patients into “risky” and “non-risky” categories has potential clinical implications, our findings should be regarded as hypothesis-generating, and randomized prospective studies are required before this strategy can be incorporated into clinical guidelines.

The dynamic shifts depicted in **Fig. 2** highlight both the promise and the constraints of serial 4D-flow monitoring. On the one hand, the observation that PVST events occurred exclusively under risky hemodynamics suggests a practical pathway: initiate or maintain anticoagulation when a risky state is present and consider cessation once a stable non-risky state is consistently observed. On the other hand, the occurrence of late PVST after a transition from non-risky to risky hemodynamics in an individual case underscores the need for longitudinal surveillance and cautions against over-reliance on a single time-point classification. Moreover, while **Fig. 3** supports the superior diagnostic performance of the composite risk equation over PV stump length, our sample size and clustered measurements preclude causal inference and definitive management thresholds; thus, prospective studies with standardized imaging intervals are required before routine clinical adoption.

In some patients, hemodynamic parameters shifted from risky to non-risky conditions after anticoagulation. However, it remains uncertain whether this improvement was attributable to the therapy itself or to the natural stabilization of blood flow following postoperative remodeling. We speculate that anticoagulation mainly prevents embolic events, while long-term normalization of hemodynamics may be driven primarily by anatomical adaptation. Further studies with prospective and standardized follow-up are required to clarify these mechanisms.

Once PVST is identified on enhanced CT, anticoagulant therapy is generally recommended to prevent vital organ embolisms. However, PVST can recur after its disappearance following anticoagulant therapy. Therefore, the optimal timing of anticoagulant discontinuation remains unclear. Based on the current study results, 4D flow MRI can help identify patients who require anticoagulant therapy.

Several limitations associated with the present study warrant mention. First, the sample size was small (n = 11). Therefore, our analysis should be considered exploratory. Nevertheless, the consistency between risky hemodynamics and the occurrence of PVST across multiple time points provides valuable mechanistic insights. Future multicenter, prospective studies with larger cohorts are warranted to validate the predictive performance of our hemodynamic risk model. Second, late postoperative 4D flow MRI was performed at the time of each patient’s clinical follow-up visit, resulting in variability in the interval between surgery and imaging (range: 3–14 months). This heterogeneity may have influenced the observed temporal changes in hemodynamic parameters, as both anatomical remodeling after lobectomy and anticoagulation therapy could alter flow dynamics. The impact of such variability should be interpreted with caution. Third, although reproducibility was confirmed in a subset of cases, further multicenter validation will be required to ensure inter-operator generalizability of SD and EL measurements. Finally, patients with atrial fibrillation were excluded because 4D flow MRI requires regular sinus rhythm. Although this was methodologically unavoidable, it limits the applicability of our findings to the broader clinical population, as atrial fibrillation itself is a known risk factor for left atrial thrombus formation. This represents an important limitation that future technical developments in MRI may help to overcome.

## Conclusion

Our results suggest that late-postoperative hemodynamic assessment, as well as early postoperative assessment, is useful for identifying patients at high risk of PVST. Late postoperative hemodynamic assessments may contribute to determining the timing of anticoagulant discontinuation.

## Declarations

### Data Availability

The data that support the findings of this study are available from the corresponding author upon reasonable request.
